# Caffeine and MDMA (Ecstasy) Exacerbate ER Stress Triggered by Hyperthermia

**DOI:** 10.3390/ijms23041974

**Published:** 2022-02-10

**Authors:** Kathleen A. Trychta, Brandon K. Harvey

**Affiliations:** Molecular Mechanisms of Cellular Stress and Inflammation Section, Intramural Research Program, National Institute on Drug Abuse, National Institutes of Health, Baltimore, MD 21224, USA

**Keywords:** ER exodosis, hyperthermia, caffeine, MDMA, KDEL receptor

## Abstract

Drugs of abuse can cause local and systemic hyperthermia, a known trigger of endoplasmic reticulum (ER) stress and the unfolded protein response (UPR). Another trigger of ER stress and UPR is ER calcium depletion, which causes ER exodosis, the secretion of ER-resident proteins. In rodent models, club drugs such as 3,4-methylenedioxymethamphetamine (MDMA, ‘ecstasy’) can create hyperthermic conditions in the brain and cause toxicity that is affected by the environmental temperature and the presence of other drugs, such as caffeine. In human studies, MDMA stimulated an acute, dose-dependent increase in core body temperature, but an examination of caffeine and MDMA in combination remains a topic for clinical research. Here we examine the secretion of ER-resident proteins and activation of the UPR under combined exposure to MDMA and caffeine in a cellular model of hyperthermia. We show that hyperthermia triggers the secretion of normally ER-resident proteins, and that this aberrant protein secretion is potentiated by the presence of MDMA, caffeine, or a combination of the two drugs. Hyperthermia activates the UPR but the addition of MDMA or caffeine does not alter the canonical UPR gene expression despite the drug effects on ER exodosis of UPR-related proteins. One exception was increased *BiP/GRP78* mRNA levels in MDMA-treated cells exposed to hyperthermia. These findings suggest that club drug use under hyperthermic conditions exacerbates disruption of ER proteostasis, contributing to cellular toxicity.

## 1. Introduction

Club drugs, or “rave” drugs, are a class of recreational drugs associated with the location of usage, such as nightclubs, raves, and dance parties. In these environments, the combination of a warm atmosphere, overcrowding, and dancing can contribute to elevated body temperature, or hyperthermia, which can negatively impact the body’s reaction to substances of abuse. For example, the combination of hyperthermic environmental conditions and one of the most widely used club drugs, 3,4-methylenedioxymethamphetamine (MDMA, ‘ecstasy’), can be lethal in humans [1,2]. Moreover, MDMA is associated with drug-induced hyperthermia and consumption of the drug can itself cause hyperthermia in humans [3,4,5,6]. The potentiation of MDMA-induced hyperthermia by social interaction and warm ambient temperature has been recreated in rodent models [7]. Consumption of other stimulants, such as caffeine, has been shown to promote hyperthermia and the toxicity associated with MDMA [8]. Caffeine is commonly present in drinks consumed with club drugs and is also used as a drug additive. The interplay between increased ambient temperature, drug usage, and cellular proteostasis has not been well documented.

Human body temperature is normally maintained at 37 °C, with fluctuations of only a degree or two being well tolerated, and temperatures that reach 40 °C or higher generally constituting hyperthermia. The central nervous system is vulnerable to hyperthermia and increased temperatures have been linked to cognitive dysfunction, seizures, and loss of consciousness, as well as exacerbating poor outcomes following brain injury (i.e., traumatic brain injury, subarachnoid hemorrhage, stroke, and substance abuse) [3,9,10,11]. At a cellular level, as temperatures rise above 37 °C the number of unfolded proteins, protein aggregates, and denatured proteins increases [12,13]. Under normal conditions, when the cell detects misfolded proteins, the unfolded protein response (UPR) is activated to promote the degradation of misfolded proteins, diminish overall protein production, and upregulate molecular chaperones to restore protein folding [14,15]. While hyperthermia triggers the UPR, this adaptive response does not always prevent cell death [16,17,18,19]. Furthermore, elevated temperatures lead to decreased chaperone activity and some chaperones can function as proteases at increased temperatures, preventing the endoplasmic reticulum (ER) from re-establishing protein homeostasis [20]. Hyperthermic disruptions to ER homeostasis have the potential to affect intrinsic ER functions such as protein processing and trafficking, lipid and carbohydrate metabolism, and drug detoxification as these ER processes rely on the presence and activity of ER luminal proteins [21,22,23,24]. 

Studies of ER-resident proteins have identified carboxy-terminal peptide sequences referred to as ER retention sequences (ERS) that localize proteins to the ER lumen [25,26,27,28,29]. If an ERS-containing protein escapes the ER lumen, the ERS sequence is recognized by KDEL receptors in the Golgi and the KDEL receptors interact with the protein to facilitate its retrograde transport back to the ER [25,30]. Under certain stress conditions, ERS-containing proteins are secreted from the cell [31,32,33,34,35]. A redistribution of ERS proteins en masse from the ER lumen to the extracellular space was observed in response to both ER calcium depletion and oxygen-glucose deprivation [36]. This phenomenon, termed ER exodosis, refers to the departure of a resident protein from its organelle under pathophysiological conditions. Given the previously described links between hyperthermia and both ER stress and changes in cellular calcium, hyperthermia represents a putative trigger of ER exodosis [16,37]. 

Here we investigate the effects of environment-induced hyperthermia on ER proteostasis in the presence and absence of MDMA and caffeine. We show for the first time that hyperthermia causes the secretion of ER-resident proteins, or ER exodosis. We find that both MDMA and caffeine exacerbate ER exodosis under hyperthermic conditions in both a human neuronal cell line and rodent primary cortical neurons. Our findings identify a cellular mechanism impacted by hyperthermia and exposure to the drugs MDMA and caffeine. The therapeutic potential of preventing ER exodosis in the treatment of club drug toxicity and hyperthermia is discussed. 

## 2. Results

### 2.1. Hyperthermia Triggers the Secretion of ER-Resident Proteins

**Figure 1:**
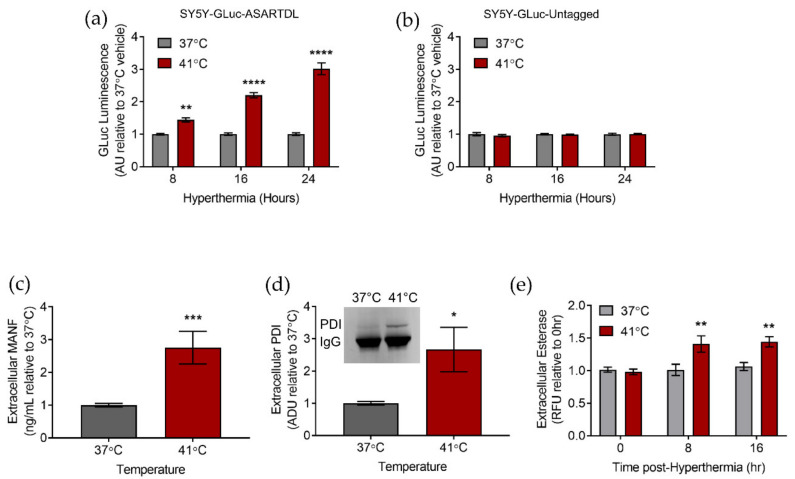
ER-resident protein secretion is triggered by hyperthermia. (**a**,**b**) GLuc (arbitrary units: AU) in the media from SH-SY5Y cells stably expressing (**a**) GLuc-ASARTDL or (**b**) GLuc-Untagged after an 8 h, 16 h, or 24 h incubation at 37 °C or 41 °C (mean ± SEM, *n* = 16, two-way ANOVA with Sidak’s multiple comparisons, ** *p* < 0.01 and **** *p* < 0.0001 37 °C vs. 41 °C). (**c**) Fold change in extracellular MANF from SH-SY5Y cells following a 24 h incubation at 37 °C or 41 °C (mean ± SEM, *n* = 5, *t*-test, *** *p* < 0.001). (**d**) Fold change in arbitrary density units (ADU) of immunoprecipitated PDI (representative blot shown) from media of SH-SY5Y cells following incubation at 37 °C or 41 °C (mean ± SEM, *n* = 3, *t*-test, * *p* < 0.05). (**e**) SH-SY5Y cells were exposed to 37 °C or 41 °C for 8 h or 16 h. Fluorescent esterase assay of cell culture media presented as a fold change in raw fluorescent units (RFU) (mean ± SEM, *n* = 12, two-way ANOVA with Sidak’s multiple comparisons, ** *p* < 0.01 37 °C vs. 41 °C).

In order to study the secretion of ER-resident proteins in a model of hyperthermia, we used a *Gaussia* luciferase (GLuc) protein with an ERS. This GLuc with an ASARTDL (Ala-Ser-Ala-Arg-Thr-Asp-Leu) C-terminal ERS was previously shown to localize to the ER lumen and act as a marker of ER exodosis [35,36]. This GLuc reporter allowed us to easily determine whether or not hyperthermia was a potential trigger for ER-resident protein secretion. As we sought to examine the implications of hyperthermia in the brain, we used the human SH-SY5Y neuroblastoma cell line. Our lab previously created and characterized SH-SY5Y cell lines that stably express the GLuc-ASARTDL reporter or a constitutively secreted GLuc reporter with no ERS (GLuc-Untagged). SH-SY5Y cells have been previously used in hyperthermia models to study neurotoxicity; however, none of the previous studies examined ER-resident protein secretion as a potential outcome of hyperthermia [38,39]. We discovered that following hyperthermia there was increased secretion of GLuc-ASARTDL whereas there was no change in GLuc-Untagged secretion (Figure 1a,b). Prolonged exposure to hyperthermia caused a decrease in cell viability (indicated by ATP levels) in both cell lines, consistent with results seen previously in SH-SY5Y (Figure S1a,b) [35,40].

To determine whether the increased secretion of GLuc-ASARTDL was reflective of the behavior of endogenous ER-resident proteins, we examined the extracellular levels of three different endogenous ERS-containing proteins (MANF, PDI, and esterases). The extracellular presence of each protein was monitored using a different type of assay and was specific to the protein of interest to minimize any potential off-target effects and demonstrate that our GLuc reporter of ER exodosis mirrors the behavior of endogenous ER-resident proteins. For each of these ER-resident proteins, extracellular protein levels were increased following hyperthermia, suggesting that hyperthermia leads to ER exodosis in which there is a mass redistribution of ERS-containing ER-resident proteins into the extracellular space following an insult (Figure 1c–e). In order to expand our study into another cell type that may more closely mimic an in vivo environment and physiology, we further examined the effects of hyperthermia in rat primary cortical neurons (PCNs) by transducing PCNs with an AAV vector expressing GLuc-ASARTDL. Similar to what was observed in SH-SY5Y cells, an increase in extracellular GLuc-ASARTDL was observed following hyperthermia in PCNs (Figure S1c). No such increase in GLuc was observed in PCNs transduced with GLuc-Untagged, although cells exhibited comparable hyperthermia-induced decreases in ATP levels regardless of the reporter used (Figure S1d–f). 

### 2.2. Manipulating ER Calcium Alters Hyperthermia-Induced ER Exodosis

Hyperthermia has a complex etiology with some links to changes in intracellular calcium [17,41]. In order for the ER to carry out its myriad of functions, luminal calcium concentrations must be tightly controlled. Under basal conditions, the sarco-/endoplasmic reticulum calcium ATPase (SERCA) pumps calcium from the cytoplasm into the ER while the ryanodine receptor (RyR) and inositol 1,4,5-triphosphate receptor (IP3R) allow for calcium efflux [42,43,44]. Reductions in ER calcium are associated with ER-resident protein secretion [32,33,34,36]. In our hyperthermia model, treatment with thapsigargin, a SERCA inhibitor, potentiated the release of GLuc-ASARTDL in hyperthermic conditions (Figure S2a). To determine whether this effect occurred following other forms of ER stress, we treated cells with tunicamycin. Both thapsigargin and tunicamycin are known to cause ER stress, with thapsigargin inhibiting ER calcium uptake and tunicamycin acting as an N-linked glycosylation inhibitor, and were used at doses previously shown to induce comparable levels of ER stress [34,45,46]. Only thapsigargin enhanced GLuc-ASARTDL secretion in hyperthermic conditions (Figure S2b). Treatment with tunicamycin caused no change in GLuc-ASARTDL levels when compared to vehicle-treated cells, suggesting that in hyperthermic conditions GLuc-ASARTDL secretion is further increased following changes in ER calcium but not changes to ER stress in general (Figure S2b). 

**Figure 2:**
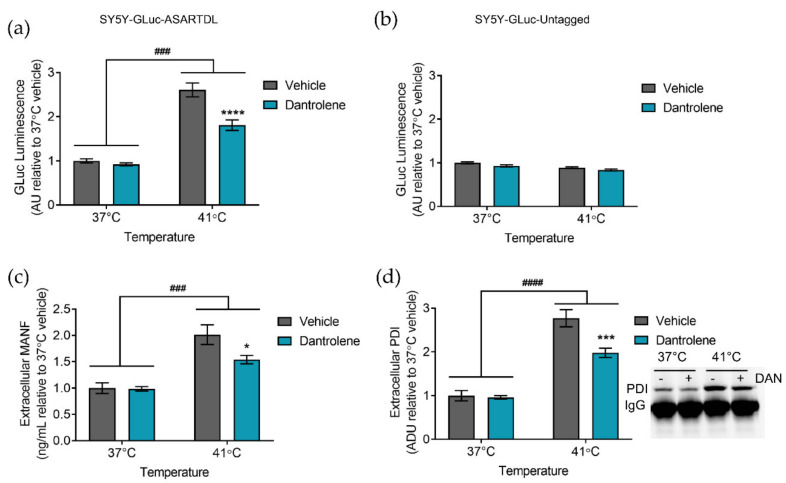
Blocking ER calcium efflux attenuates hyperthermia-induced ER-resident protein secretion. (**a**,**b**) GLuc (arbitrary units: AU) in the media from SH-SY5Y cells stably expressing either (**a**) GLuc-ASARTDL or (**b**) GLuc-Untagged after a 16 h pre-treatment with 3 µM dantrolene followed by a 24 h incubation at 37 °C or 41 °C (mean ± SEM, *n* = 12, two-way ANOVA with Dunnett’s multiple comparisons, ### *p* < 0.001 37 °C vs. 41 °C, **** *p* < 0.0001 vehicle vs. dantrolene). (**c**) Fold change in extracellular MANF from SH-SY5Y cells following a 16 h pre-treatment with 3 µM dantrolene and 24 h exposure to 37 °C or 41 °C (mean ± SEM, *n* = 3, two-way ANOVA with Sidak’s multiple comparisons, ### *p* < 0.001 37 °C vs. 41 °C, * *p* < 0.05 vehicle vs. dantrolene). (**d**) Fold change in arbitrary density units (ADU) of immunoprecipitated PDI (representative blot shown) from the media of SH-SY5Y cells following a 16 h pre-treatment with 3 µM dantrolene and 24 h exposure to 37 °C or 41 °C (mean ± SEM, *n* = 6, two-way ANOVA with Sidak’s multiple comparisons, #### *p* < 0.0001 37 °C vs. 41 °C, *** *p* < 0.001 vehicle vs. dantrolene).

In addition to altering calcium influx into the ER, we also examined how blocking calcium efflux from the ER affects ER exodosis. Dantrolene, a RyR antagonist, can attenuate ER calcium depletion and decrease ER-resident protein secretion [34,35,36,47]. In our model of hyperthermia, treating with dantrolene attenuated the secretion of GLuc-ASARTDL observed following hyperthermia, while having no effect on the secretion of GLuc-Untagged (Figure 2a,b and Figure S2c). Treatment with dantrolene did not affect cellular ATP levels (Figure S2d). An examination of MANF and PDI, two endogenous ERS-containing proteins, found an increase in extracellular protein levels following hyperthermia, which was diminished by treatment with dantrolene (Figure 2c,d). The behavior of endogenous MANF and PDI mirrored the results seen with the GLuc-ASARTDL reporter, supporting a model in which hyperthermia leads to ER exodosis that can be partially blunted by stabilizing ER calcium. Despite the ability of dantrolene to attenuate hyperthermia-induced secretion in SH-SY5Y, dantrolene was unable to reduce GLuc-ASARTDL secretion in PCNs or affect ATP levels (Figure S2e,f). Because dantrolene acts primarily on the RyR1 and RyR3 isoforms of the RyR, and neurons primarily express RyR2, we expanded our analysis to look at modulators of IP3R activity [48,49]. Stabilizing ER calcium by inhibiting IP3R with 2-APB reduced extracellular GLuc levels in PCNs transduced with GLuc-ASARTDL and exposed to hyperthermia, while no effect was seen on cells transduced with GLuc-Untagged (Figure S2g) [50]. 2-APB did not significantly affect cell viability (Figure S2h). Similar to the results seen with GLuc-ASARTDL, extracellular levels of the endogenous ERS-containing protein PDI were increased following hyperthermia, with reduced levels observed in 2-APB-treated cells (Figure S2i). Overall, these pharmacological manipulations were used to highlight the association we observed between ER calcium depletion and hyperthermia as triggers of ER exodosis. 

### 2.3. Hyperthermia Affects the Unfolded Protein Response

**Figure 3:**
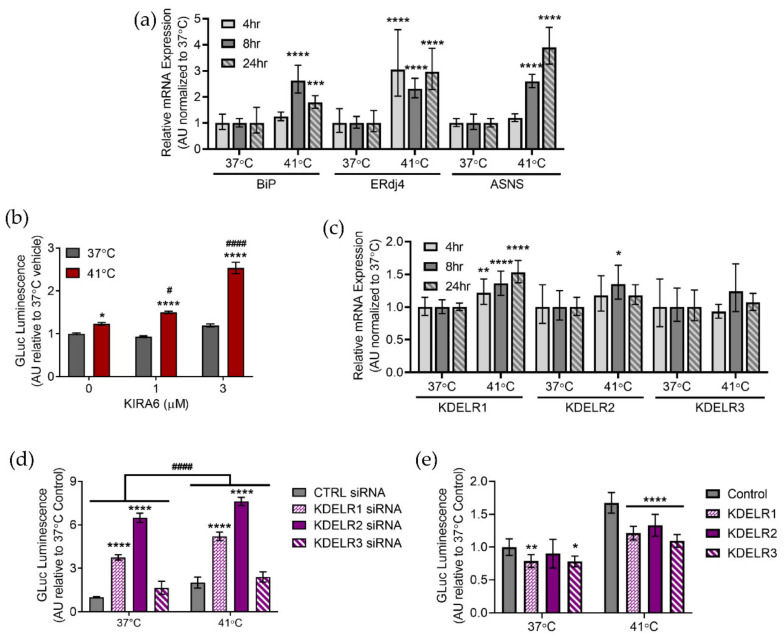
The UPR and KDEL receptors in hyperthermic conditions. (**a**) *BiP*, *ERdj4*, and *ASNS* mRNA levels analyzed with real-time RT-qPCR after a 4, 8, or 24 h exposure to 37 °C or 41 °C. Data are shown as relative mRNA expression (arbitrary units: AU) relative to control (2^−ddCq^ ± upper and lower limits, *n* = 8, two-way ANOVA with Sidak’s multiple comparisons, *** *p* < 0.001 and **** *p* < 0.0001 37 °C vs. 41 °C). (**b**) GLuc (arbitrary units: AU) in the media from SH-SY5Y cells stably expressing GLuc-ASARTDL after a 1 h treatment with vehicle or KIRA6 (1 µM or 3 µM) followed by a 24 h incubation at 37 °C or 41 °C (mean ± SEM, *n* = 48, two-way ANOVA with Tukey’s multiple comparisons, * *p* < 0.05 and **** *p* < 0.0001 37 °C vs. 41 °C, # *p* < 0.01 and #### *p* < 0.0001 vehicle vs. KIRA6). (**c**) *KDELR1*, *KDELR2*, and *KDELR3* mRNA levels analyzed with real-time RT-qPCR after a 4, 8, or 24 h exposure to 37 °C or 41 °C. Data are shown as relative mRNA expression (arbitrary units: AU) relative to control (2^−ddCq^ ± upper and lower limits, *n* = 8, two-way ANOVA with Sidak’s multiple comparisons, * *p* < 0.05, ** *p* < 0.01, and **** *p* < 0.0001 37 °C vs. 41 °C). (**d**) GLuc (arbitrary units: AU) in media following transfection of SH-SY5Y cells stably expressing GLuc-ASARTDL with 10 nM KDEL receptor siRNA (mean ± SEM, *n* = 12, two-way ANOVA with Dunnett’s multiple comparisons, #### *p* < 0.0001 37 °C vs. 41 °C, **** *p* < 0.0001 control vs. KDELR siRNA). (**e**) GLuc (arbitrary units: AU) in media following transduction of SH-SY5Y cells stably expressing GLuc-ASARTDL with lentiviral KDEL receptors and a 24 h exposure to 37 °C or 41 °C (mean ± SEM, *n* = 12, two-way ANOVA with Dunnett’s multiple comparisons, *p* < 0.0001 37 °C vs. 41 °C, * *p* < 0.05, ** *p* < 0.01, and **** *p* < 0.0001 control vs. LV-KDELR).

The unfolded protein response (UPR) is an adaptive cellular response to ER stress that seeks to restore proteostasis and maintain cell viability. Previous studies indicate that hyperthermia provokes the UPR and we show here that incubation at 41 °C results in upregulation of the UPR target genes *BiP*, *ERdj4*, and *ASNS* (Figure 3a) [16,18]. By examining these three genes we were able to obtain a representative readout of the UPR as *BiP*, *ERdj4*, and *ASNS* are activated by the ATF6, IRE1α/XBP1, and PERK UPR pathways, respectively [51,52]. There appears to be a link between UPR activation and length of exposure to hyperthermic conditions as all three UPR target genes are upregulated at 8 h and 24 h, but only *ERdj4* is upregulated at 4 h (Figure 3a). *ERdj4* is a target gene of the IRE1α/XBP1 prong of the UPR. As previous work from our lab demonstrated that ER exodosis is modulated by the KDEL receptor retrieval pathway and that *KDELR2* and *KDELR3* are UPR responsive genes regulated by XBP1 we sought to further examine these findings in our model of hyperthermia [36]. In our model, inhibiting IRE1α kinase activity with KIRA6 potentiated hyperthermia-induced GLuc-ASARTDL secretion, but had no effect on GLuc-ASARTDL in normothermic conditions in both SH-SY5Y cells and PCNs (Figure 3b and Figure S3a) [53]. Treatment with KIRA6 did not increase extracellular GLuc levels in GLuc-Untagged cells (Figure S2b,c). Despite the observed upregulation of *ERdj4* and increased ER exodosis following treatment with KIRA6, mRNA levels of the KDEL receptors remained relatively unchanged with only *KDELR1* being minimally upregulated following hyperthermia (Figure 3c). However, knocking down KDEL receptors increased GLuc-ASARTDL and MANF release indicating that the KDEL receptors still play an important role in hyperthermia-induced ER exodosis (Figure 3d and Figure S3d). GLuc-Untagged secretion was not affected by KDEL receptor knockdown (Figure S3e). Conversely, overexpressing KDEL receptors decreased GLuc-ASARTDL secretion following hyperthermia (Figure 3e). By both knocking down and overexpressing KDEL receptors we were able to establish the function of the KDEL receptor retrieval pathway in ER exodosis related to hyperthermia. Taken together these results indicate that hyperthermia triggers the UPR and that ER exodosis is sensitive to manipulations of the KDEL receptor retrieval pathway.

### 2.4. Caffeine and MDMA Affect ER Responses to Hyperthermia

**Figure 4:**
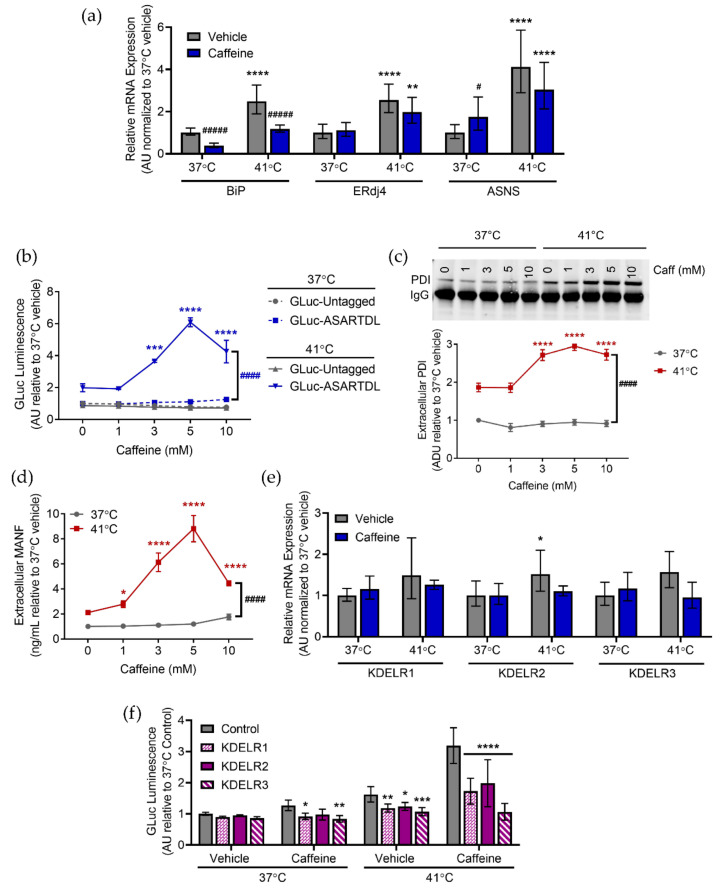
Caffeine changes cellular responses to hyperthermia. (**a**) *BiP*, *ERdj4*, and *ASNS* mRNA levels analyzed with real-time RT-qPCR after treatment with vehicle or 5 mM caffeine and an 8 h exposure to 37 °C or 41 °C. Data are shown as relative mRNA expression (arbitrary units: AU) relative to control (2^−ddCq^ ± upper and lower limits, *n* = 8, two-way ANOVA with Sidak’s multiple comparisons, ** *p* < 0.01 and **** *p* < 0.0001 37 °C vs. 41 °C, # *p* < 0.05, ##### *p* < 0.001 vehicle vs. caffeine). (**b**) GLuc (arbitrary units: AU) in the media from SH-SY5Y cells stably expressing GLuc-ASARTDL or GLuc-Untagged after treatment with vehicle or caffeine and a 24 h incubation at 37 °C or 41 °C (mean ± SEM, *n* = 26, two-way ANOVA with Tukey’s multiple comparisons, #### *p* < 0.0001 37 °C vs. 41 °C, *** *p* < 0.001 and **** *p* < 0.0001 vehicle vs. caffeine). (**c**,**d**) Fold change in (**c**) arbitrary density units (ADU) of immunoprecipitated PDI (representative blot shown) or (**d**) MANF in media from SH-SY5Y cells treated with vehicle or caffeine and incubated for 24 h at 37 °C or 41 °C (mean ± SEM, *n* = 6, two-way ANOVA with Dunnett’s multiple comparisons, #### *p* < 0.0001 37 °C vs. 41 °C, * *p* < 0.05 and **** *p* < 0.0001 vehicle vs. caffeine). (**e**) *KDELR1*, *KDELR2*, and *KDELR3* mRNA levels analyzed with real-time RT-qPCR after treatment with vehicle or 5 mM caffeine and an 8 h exposure to 37 °C or 41 °C. Data are shown as relative mRNA expression (arbitrary units: AU) relative to control (2^−ddCq^ ± upper and lower limits, *n* = 8, two-way ANOVA with Sidak’s multiple comparisons, * *p* < 0.05 37 °C vs. 41 °C). (**f**) GLuc (arbitrary units: AU)in media following transduction of SH-SY5Y cells stably expressing GLuc-ASARTDL with lentiviral KDEL receptors and treatment with vehicle or 5 mM caffeine and a 24 h exposure to 41 °C (mean ± SEM, *n* = 9, two-way ANOVA with Dunnett’s multiple comparisons, *p* < 0.0001 37 °C vs. 41 °C, * *p* < 0.05, ** *p* < 0.01, *** *p* < 0.001, and **** *p* < 0.0001 control vs. LV-KDELR).

Caffeine is one of the most widely used drugs in the world and it is known to affect ER calcium by acting as a RyR agonist. We previously showed that GLuc-ASARTDL in the media was increased following a 72 h incubation with 5 and 10 mM caffeine [35]. Therefore, we sought to examine the combined effects of a more acute caffeine treatment and hyperthermia on UPR activation and ER exodosis. As seen previously, hyperthermia triggered a UPR response in cells. Caffeine treatment did not augment UPR activation in hyperthermic cells but did result in decreased *BiP* mRNA levels as well as increased *ASNS* mRNA levels at normal temperatures (Figure 4a). Caffeine alone did not significantly change GLuc-ASARTDL secretion at normal temperatures, but caffeine did trigger a robust increase in secretion under hyperthermic conditions (Figure 4b). GLuc-Untagged secretion was unaffected by hyperthermia or caffeine (Figure 4b). Caffeine at the highest dose (10 mM) was associated with reduced cellular ATP levels (Figure S4a). In accordance with the GLuc-ASARTDL data, both extracellular PDI and MANF were increased following hyperthermia and caffeine caused a significant, dose-dependent increase in extracellular levels of both endogenous ERS proteins (Figure 4c,d). Similar findings were seen in PCNs with caffeine augmenting hyperthermia-induced ER exodosis and decreasing ATP levels (Figure S4b–d). As demonstrated previously, treating with dantrolene reduced GLuc-ASARTDL secretion caused by hyperthermia, but only a minimal effect was seen when cells were exposed to both hyperthermia and caffeine, perhaps because both caffeine and dantrolene act on the RyR or the effects of caffeine on other signaling pathways exacerbate hyperthermia-induced exodosis (Figure S4e). GLuc-Untagged secretion was not affected by hyperthermia, caffeine, or dantrolene and ATP levels were only affected by hyperthermia (Figure S4f,g). Neither hyperthermia nor caffeine changed KDEL receptor mRNA levels, but overexpression of KDEL receptors inhibited hyperthermia and caffeine-induced GLuc-ASARTDL secretion (Figure 4e,f). 

**Figure 5:**
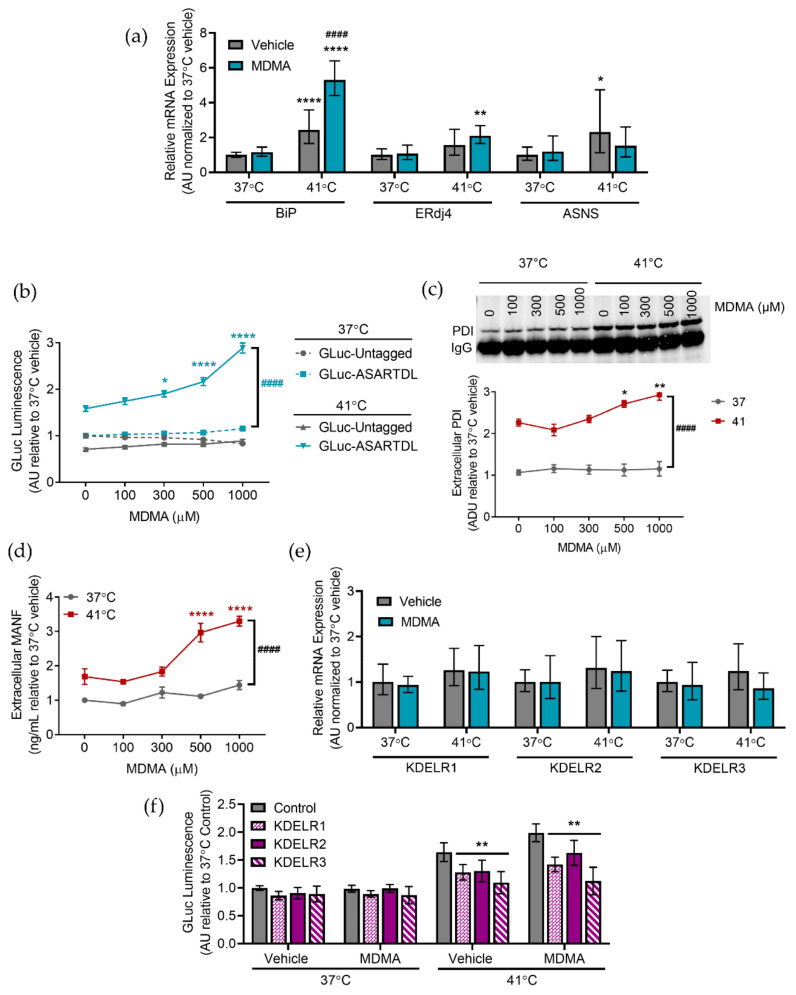
MDMA changes cellular responses to hyperthermia. (**a**) *BiP*, *ERdj4*, and *ASNS* mRNA levels analyzed with real-time RT-qPCR after treatment with vehicle or 1 mM MDMA and an 8 h exposure to 37 °C or 41 °C. Data are shown as relative mRNA expression (arbitrary units: AU) relative to control (2^−ddCq^ ± upper and lower limits, *n* = 8, two-way ANOVA with Sidak’s multiple comparisons, * *p* < 0.05, ** *p* < 0.01 and **** *p* < 0.0001 37 °C vs. 41 °C, #### *p* < 0.001 vehicle vs. caffeine). (**b**) GLuc (arbitrary units: AU) in the media from SH-SY5Y cells stably expressing GLuc-ASARTDL or GLuc-Untagged after treatment with vehicle or MDMA and a 24 h incubation at 37 °C or 41 °C (mean ± SEM, *n* = 26, two-way ANOVA with Tukey’s multiple comparisons, #### *p* < 0.0001 37 °C vs. 41 °C, * *p* < 0.05, **** *p* < 0.0001 vehicle vs. caffeine). (**c**,**d**) Fold change in (**c**) arbitrary density units (ADU) of immunoprecipitated PDI (representative blot shown) or (**d**) MANF in media from SH-SY5Y cells treated with vehicle or MDMA and incubated for 24 h at 37 °C or 41 °C (mean ± SEM, *n* = 6, two-way ANOVA with Dunnett’s multiple comparisons, #### *p* < 0.0001 37 °C vs. 41 °C, * *p* < 0.05, ** *p* < 0.01, and **** *p* < 0.0001 vehicle vs. caffeine). (**e**) *KDELR1*, *KDELR2*, and *KDELR3* mRNA levels analyzed with real-time RT-qPCR after treatment with vehicle or 1 mM MDMA and an 8 h exposure to 37 °C or 41 °C. Data are shown as relative mRNA expression (arbitrary units: AU) relative to control (2^−ddCq^ ± upper and lower limits, *n* = 8, two-way ANOVA with Sidak’s multiple comparisons). (**f**) GLuc (arbitrary units: AU) in media following transduction of SH-SY5Y cells stably expressing GLuc-ASARTDL with lentiviral KDEL receptors and treatment with vehicle or 1 mM MDMA and a 24 h exposure to 37 °C or 41 °C (mean ± SEM, *n* = 9, two-way ANOVA with Dunnett’s multiple comparisons, *p* < 0.0001 37 °C vs. 41 °C, ** *p* < 0.01 control vs. LV-KDELR).

The drug of abuse, 3,4-methylenedioxymethamphetamine (MDMA), can have more severe effects in certain environmental conditions, including elevated temperatures [54]. SH-SY5Y cells have been used previously to study the effects of MDMA, so we wanted to examine whether MDMA affected ER stress and exodosis [55,56]. We observed a marked increase in *BiP* expression in hyperthermic conditions when MDMA was present, although *ERdj4* and *ASNS* levels were not changed by the presence of MDMA (Figure 5a). Following exposure to MDMA in hyperthermic conditions, we found increased GLuc-ASARTDL secretion beyond that seen in vehicle conditions (Figure 5b). MDMA did not change GLuc-ASARTDL secretion in normothermic controls and the control reporter, GLuc-Untagged, was not increased by hyperthermia or MDMA (Figure 5b). MDMA also caused a decrease in cellular ATP at higher doses under hyperthermic conditions (Figure S5a). Extracellular levels of the ERS-containing proteins PDI and MANF were increased following hyperthermia and were more highly secreted with increasing concentrations of MDMA, suggesting that MDMA increases the secretion of ERS-containing proteins, but only in hyperthermic conditions (Figure 5c,d). Studies in PCNs revealed a similar pattern, with MDMA exacerbating ER-resident protein secretion and decreasing cell viability at elevated temperatures (Figure S5b–d). Stabilizing ER calcium by treatingwith dantrolene reduced GLuc-ASARTDL secretion resulting from hyperthermia and MDMA, while GLuc-Untagged secretion was unchanged and ATP levels were only affected by hyperthermia (Figure S5e–g). KDEL receptor expression was not changed by increased temperature or MDMA, but the overexpression of KDEL receptors attenuated GLuc-ASARTDL secretion following hyperthermia and MDMA treatment (Figure 5e,f).

**Figure 6:**
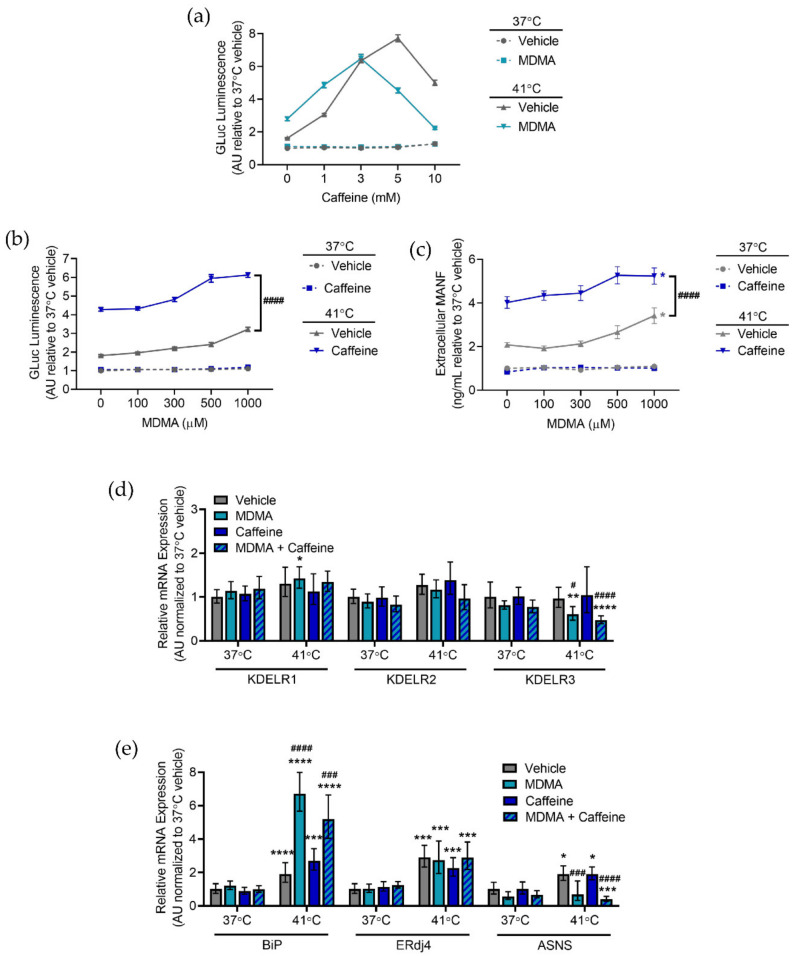
Caffeine and MDMA in combination affect hyperthermia-induced cellular responses. (**a**) GLuc (arbitrary units: AU) in the media from SH-SY5Y cells stably expressing GLuc-ASARTDL after treatment with vehicle or 500 µM MDMA in combination with a dose response of caffeine and incubation at 37 °C or 41 °C for 24 h (mean ± SEM, *n* = 24). (**b**) GLuc (arbitrary units: AU) in the media from SH-SY5Y cells stably expressing GLuc-ASARTDL after treatment with vehicle or 1 mM caffeine in combination with a dose response of MDMA and incubation at 37 °C or 41 °C for 24 h (mean ± SEM, *n* = 24, three-way ANOVA with slice decomposition, #### *p* < 0.0001 vehicle vs. caffeine at 41 °C). (**c**) MANF in media from SH-SY5Y cells treated with vehicle or 1 mM caffeine in combination with a dose response of MDMA and incubation at 37 °C or 41 °C for 24 h (mean ± SEM, *n* = 6, three-way ANOVA with slice decomposition, * *p* < 0.05 vehicle vs. MDMA at 41 °C, #### *p* < 0.0001 vehicle vs. caffeine at 41 °C. (**d**) *KDELR1*, *KDELR2*, and *KDELR3* mRNA levels analyzed with real-time RT-qPCR after treatment with vehicle, 1 mM MDMA, 1 mM caffeine, or a combination of 1 mM MDMA and 1 mM caffeine and an 8 h exposure to 37 °C or 41 °C. Data are shown as relative mRNA expression (arbitrary units: AU) relative to control (2^−ddCq^ ± upper and lower limits, *n* = 8, two-way ANOVA with Sidak’s multiple comparisons, * *p* < 0.05, ** *p* < 0.01 and **** *p* < 0.0001 37 °C vs. 41 °C, # *p* < 0.05 and #### *p* < 0.001 vehicle vs. drug treatment). (**e**) *BiP*, *ERdj4*, and *ASNS* mRNA levels analyzed with real-time RT-qPCR after treatment with vehicle, 1 mM MDMA, 1 mM caffeine, or a combination of 1 mM MDMA and 1 mM caffeine and an 8 h exposure to 37 °C or 41 °C. Data are shown as relative mRNA expression (arbitrary units) relative to control (2^−ddCq^ ± upper and lower limits, *n* = 8, two-way ANOVA with Sidak’s multiple comparisons, * *p* < 0.05, *** *p* < 0.001 and **** *p* < 0.0001 37 °C vs. 41 °C, ### *p* < 0.001 and #### *p* < 0.0001 vehicle vs. drug treatment).

From a club drug perspective, the combination of caffeine, MDMA, and hyperthermia warrants further study. Clubs are often crowded venues with dancing, both of which increase the ambient temperature of the room. MDMA is a common club drug and is often combined with caffeine in the form of drinks containing caffeine. MDMA is also one of the most adulterated drugs, and caffeine is one of the substances used to cut MDMA. Treating SH-SY5Y with MDMA resulted in a leftward shift of a caffeine dose response with an increase in hyperthermia-induced GLuc-ASARTDL secretion observed in cells treated with MDMA and 1 mM caffeine (Figure 6a). At no dose of caffeine was extracellular GLuc-ASARTDL increased in the normal temperature control (37 °C) regardless of whether the cells were treated with MDMA (Figure 6a). Furthermore, no condition changed GLuc-Untagged secretion (Figure S6a). As observed previously, increasing doses of caffeine diminished cellular ATP (Figure S6b). Using 1 mM caffeine in combination with a dose response of MDMA revealed a significant potentiation of GLuc-ASARTDL hyperthermia-induced secretion with MDMA treatment (Figure 6b). The combination of caffeine, MDMA, and hyperthermia also led to increased secretion of the endogenous ERS-containing protein MANF (Figure 6c). No change in GLuc-Untagged secretion was observed and while higher doses of MDMA led to decreased cellular ATP, the presence of caffeine did not significantly exacerbate ATP loss (Figure S6c,d). No combination of temperature, MDMA, or caffeine increased KDEL receptor expression (Figure S6d). While hyperthermia did alter UPR gene expression, the combination of MDMA and caffeine did not cause *BiP*, *ERdj4*, or *ASNS* upregulation beyond that seen with either drug alone (Figure 6e). Together, these data indicate that caffeine and MDMA enhance ER-resident protein secretion during hyperthermia and that the combination of drugs further augments secretion. While MDMA increased *BiP* expression in hyperthermic conditions, other UPR markers were not affected by caffeine, MDMA, or a combination of the two drugs. 

## 3. Discussion

The lumen of the endoplasmic reticulum is the site of many critical cellular functions, such as protein folding and trafficking, lipid and carbohydrate metabolism, drug detoxification, and intracellular calcium storage. Resident proteins with ERS tails mediate these functions and are secreted when ER calcium is depleted in a process referred to as ER exodosis [36]. Following the discovery of ER exodosis, this is the first study to show that hyperthermia triggers a redistribution of the ER-resident proteome into the extracellular space. To our knowledge, this is also the first time the consequences of club drugs and hyperthermic conditions on ER proteostasis have been reported. Here, we used both an exogenous reporter of ER exodosis, GLuc-ASARTDL, and endogenous ERS proteins (MANF, PDI, and esterases) to show that hyperthermia triggered ER exodosis in both human and rodent neuronal cells. We also demonstrate that both caffeine and MDMA increase the magnitude of ER-resident protein secretion under hyperthermic conditions.

Previous findings indicate that ER exodosis and GLuc-ASARTDL secretion are linked to depletions in ER calcium [35]. Calcium within the ER lumen is usually maintained at very high concentrations, with estimations putting ER calcium concentrations at 1000 to 10,000 times greater than calcium concentrations in the cytosol [57,58,59,60,61]. Hyperthermia causes an increase in intracellular free calcium that is thought to be at least partially due to the release of calcium from internal stores such as the ER [37]. Our results implicate a role for ER calcium in hyperthermia as drugs that stabilize ER calcium (dantrolene and 2-APB) by blocking ER calcium efflux channels reduce the ER protein secretion seen at elevated temperatures. We also found that further depleting ER calcium with thapsigargin during hyperthermic conditions potentiates ER exodosis. Changes in ER calcium stores may also underlie the enhanced ER exodosis seen in both caffeine and MDMA-treated cells exposed to hyperthermia conditions. Caffeine is a documented RyR agonist that induces calcium release from intracellular ER calcium stores, while MDMA treatment is associated with increased cytosolic calcium levels resulting at least in part from depletions of intracellular ER calcium stores as blockers of ER calcium efflux (dantrolene and 2-APB) attenuate MDMA-induced cytosolic calcium increases [62,63,64]. In addition to these direct links to ER calcium stores, caffeine and MDMA are also associated with indirect processes that may affect ER calcium. Caffeine is a phosphodiesterase (PDE) inhibitor that may affect ER calcium stores through cAMP signaling. PDE normally breaks down cAMP, but caffeine blocks this process, leading to increased IP3-evoked ER calcium release [65]. MDMA stimulates serotonin release, which works through a G-protein coupled receptor pathway in which phospholipase C (PLC) is activated and hydrolyzes phosphatidylinositol biphosphate (PIP2) into IP3. IP3 can then stimulate ER calcium release through a second messenger system [66]. 

Cells treated with caffeine or MDMA and hyperthermia showed increased ER exodosis as compared to vehicle and hyperthermia-treated cells for both the human SH-SY5Y cells and rat primary cortical neurons. There were some differences in the responses at higher concentrations, where viability was most affected. For example, the highest concentration of caffeine tested (10 mM) was associated with decreased viability in both SH-SY5Y and PCNs. However, the 10 mM dose of caffeine caused further increases in hyperthermia-induced ER exodosis in PCNs, whereas the hyperthermia-induced ER exodosis at 10 mM in SH-SY5Y cells was lower than with the 5 mM caffeine dose, but still significantly higher than vehicle. The observed discrepancies in response between SH-SY5Y and primary neurons occur at high concentrations of drugs where other cellular mechanisms may be engaged. We speculate that additional downstream effects of caffeine at higher concentrations may change ER exodosis and cell viability through alternative mechanisms. For example, caffeine has indirect effects described in the paragraph above that could contribute to cell death or ER exodosis through pathways distinct from the cellular secretory pathway. Previous data also indicates that impairing other aspects of the secretory pathway, such as using brefeldin A to disrupt anterograde trafficking, can prevent ER exodosis without improving cell viability [36]. Furthermore, SH-SY5Y and PCNs may differ in their responses to caffeine due to species-related receptor-drug interactions at higher drug concentrations as well as differences in drug metabolism. For example, previous work notes that humans and rats exhibit different metabolite profiles following caffeine treatment [67]. Unlike caffeine, MDMA elicited a similar ER exodosis phenotype in SH-SY5Y and PCNs, but comparisons between caffeine and MDMA are difficult to make given the separate mechanisms of action through which the drugs work and the different pathways through which the drugs can alter cell signaling and change calcium homeostasis. Overall, the trend remains that drug treatment (caffeine or MDMA) at higher doses increases ER exodosis in hyperthermic conditions. Our results showing increased ER-resident protein secretion in hyperthermic conditions that was further enhanced by caffeine and MDMA support the need for additional studies examining the specific interplay of ER calcium stores, hyperthermia, caffeine, and MDMA in vivo.

Although the exact mechanism by which hyperthermia triggers ER-resident protein secretion remains unknown, changes in the unfolded protein response (UPR) and KDEL receptors may play a role in modulating this secretion. Prolonged exposure to hyperthermia (8 h and 24 h) was associated with upregulation of three UPR target genes. Further experimentation showed that the IRE1α pathway of the UPR was of particular importance as inhibiting IRE1α kinase activity potentiated extracellular GLuc-ASARTDL levels in hyperthermic conditions. When ERS-containing proteins leave the ER, they interact with KDEL receptors in the Golgi and are transported back to the ER [25,30]. We previously demonstrated that KDEL receptors are upregulated by ER stress following XBP1 activation [36]. In our hyperthermia model, we saw an increase in *ERdj4* expression (an XBP1 regulated gene) following hyperthermia and observed increased ER exodosis following treatment with KIRA6, a drug that prevents XBP1 activation by blocking IRE1α. Given the connection between XBP1 and KDEL receptors, we hypothesized that *KDELR2* and *KDELR3* expression may be increased in hyperthermic conditions because those two isoforms of KDEL receptors are IRE1α/XBP1 regulated. However, we observed minimal KDEL receptor upregulation following hyperthermia. Despite the lack of response, this is an important piece of data as the KDEL receptors remain an understudied part of the secretory pathway and studies examining all three isoforms are quite limited. While we observed no change in endogenous KDEL receptor mRNA levels, manipulations of KDEL receptors did affect ER exodosis. The knockdown of KDEL receptors increased ER-resident protein secretion in both normothermic and hyperthermic environments, while overexpression of KDEL receptors attenuated ER exodosis following hyperthermia. This makes KDEL receptor activation an attractive therapeutic target. Additional examinations of the UPR and KDEL receptor retrieval pathways are needed to determine precisely what role these processes play in hyperthermia and how they affect hyperthermia-induced ER exodosis. It is also worth noting that elevated temperatures change the cellular environment and are associated with decreased RNA, DNA, and protein synthesis, which may be one reason that no upregulation of KDEL receptors was observed [68]. 

The behavior of ER-resident proteins at high temperatures also needs to be taken into consideration when experimental procedures require elevations in temperature. For example, a commonly used marker of exocytic membrane trafficking is temperature sensitive, with a downshift in temperature prompting trafficking [69]. The high temperatures (around 40 °C) that cells are held at prior to the induction of trafficking have the potential to lead to ER exodosis and ER stress, which could affect the subsequently observed trafficking process. Another instance in which hyperthermia triggered ER exodosis should be considered is the use of temperature-inducible promoters [70,71]. Elevations in temperature trigger the expression of the gene of choice but could also prompt unintended changes in cellular proteostasis. 

Taken together, our data support a model in which ER-resident proteins, including some UPR proteins, are secreted from the cell in response to elevated temperatures. Our observations indicate that such a disruption of ER proteostasis may be a symptom of prolonged hyperthermia and could underlie hyperthermia-associated drug toxicity. Currently, patients with elevated body temperature are given anti-pyretics or treated non-pharmacologically with body cooling procedures (e.g., ice, cooling blankets) [72]. However, these treatments do not directly address the cellular deficits that manifest following elevated temperatures. As ER exodosis represents a novel drug-targetable cellular mechanism of dysfunction, our findings have implications for the treatment of hyperthermia-associated impairments [73]. Furthermore, the convergence of elevated temperatures with caffeine or MDMA exposure to potentiate ER exodosis constitutes a therapeutic opportunity for decreasing drug-induced cellular toxicity associated with club drug usage. In case reports of MDMA overdose, treatment with dantrolene has been part of a successful treatment paradigm, and the ability of dantrolene to promote ER proteostasis may provide a cellular explanation for this success [74,75]. Treatment options for substances of abuse remain limited, and targeting ER exodosis in hyperthermia-associated overdoses could assist in recovery. Alternatively, further augmenting the UPR with small molecular regulators of the UPR may aid in the recovery from acute overdose [76].

## 4. Materials and Methods

### 4.1. Reagents

Dantrolene (Sigma-Aldrich, St. Louis, MO, USA), thapsigargin (Sigma-Aldrich, St. Louis, MO, USA), tunicamycin (Sigma-Aldrich, St. Louis, MO, USA), dithiothreitol (DTT; Sigma-Aldrich, St. Louis, MO, USA), KIRA6 (Cayman Chemicals, Ann Arbor, MI, USA), 2-Aminoethyl diphenylborinate (2-APB; Sigma-Aldrich, St. Louis, MO, USA), caffeine (Sigma-Aldrich, St. Louis, MO, USA), 3,4-methylenedioxymethamphetamine (DL-MDMA HCl, NIH/NIDA Pharmacy, Baltimore, MD, USA).

### 4.2. SH-SY5Y Cell Culture

The creation of SH-SY5Y cells stably expressing either GLuc-ASARTDL or GLuc-Untagged was described previously [35]. Cells were maintained in Dulbecco’s Modified Eagle Medium (DMEM) + GlutaMAX, 4.5 g/L D-glucose, 110 mg/L sodium pyruvate (Thermo Fisher Scientific, Waltham, MA, USA) supplemented with 10% bovine growth serum (GE Life Sciences, Marlborough, MA, USA), 10 units/mL penicillin, and 10 μg/mL streptomycin (Thermo Fisher Scientific, Waltham, MA, USA). Cells were grown at 37 °C with 5.5% CO_2_ in a humidified incubator. Cells were plated in 96-well plates with 5 × 10^4^ cells/well or 24-well plates with 2.5 × 10^5^ cells/well. After 24 h, cells were either transferred to an incubator maintained at 41 °C or remained at 37 °C. Samples were collected after 24 h of incubation. This 24 h hyperthermia incubation time has been used in previous publications [38,39]. Incubator temperatures were monitored with a K-type thermocouple probe (Omega, Norwalk, CT, USA). 

### 4.3. Primary Cortical Neuron Cell Culture

Rat primary cortical neurons were prepared from Sprague-Dawley rats on embryonal day 15 (E15) as previously described and in accordance with National Institutes of Health Animal Care and Usage Committee guidelines [77]. Embryos of both sexes were used, and cells were plated on polyethyleneimine-coated 96-well plates with 6.0 × 10^4^ cells/well. Cells were maintained in neurobasal media (Thermo Fisher Scientific, Waltham, MA, USA) supplemented 2% B-27 (Thermo Fisher Scientific, Waltham, MA, USA) and 0.5 mM L-glutamine (Sigma, St. Louis, MO, USA) at 37 °C with 5.5% CO_2_ in a humidified incubator. A 50% media exchange was performed on days 4, 6, 8, 11, and 13. Viral transductions were performed on day 6 with 5 µL of AAV1-CaMKII-GLuc-ASARTDL (Addgene 149503, Watertown, MA, USA) or AAV1-CaMKII-GLuc-Untagged (Addgene 149502, Watertown, MA, USA) at 5.48 × 10^9^ vg/mL. On day 13, cells were either transferred to an incubator maintained at 41 °C or remained at 37 °C. Samples were collected after 24 h of incubation. This experimental paradigm was in accordance with previously published data [35]. Incubator temperatures were monitored with a K-type thermocouple probe (Omega, Norwalk, CT, USA). 

### 4.4. Gaussia Luciferase Assay

*Gaussia* luciferase (GLuc) assays were performed as described previously [35,36]. Briefly, 5 µL of cell culture media was transferred to an opaque walled plate and luciferase levels were determined using a BioTek Synergy 2 plate reader (Biotek/Agilent, Santa Clara, CA, USA) with an injector setup. Following an injection of 100 μL of 10 µm coelenterazine (Regis Technologies, Morton Grove, IL, USA) luminescence at 25 °C was measured with a sensitivity of 100, a 0.5 sec integration time, and a 5 sec delay. Data are presented as arbitrary luminescent units (AU) relative to control. 

### 4.5. ATP Assay

The Promega CellTiter-Glo Luminescent Cell Viability Assay (ATP assay, Promega, Madison, WI, USA) was used as per the manufacturer’s protocol and in accordance with previously published data [36,73]. Briefly, an equal volume of ATP substrate was added to the cell culture plate and incubated with agitation for 2 min at room temperature. After a further 10 min incubation without agitation, 100 µL of solution was transferred to an opaque-walled plate and luminescence was read using a BioTek Synergy 2 plate reader (Biotek/Agilent, Santa Clara, CA, USA). Data are presented as arbitrary luminescent units (AU).

### 4.6. PDI Immunoprecipitation

PDI immunoprecipitation was performed as described previously [36,73]. 100 µL of protein A beads (SureBeads, Bio-Rad, Hercules, CA, USA) were washed with PBS + 0.1% Tween 20 (PBS-T) and incubated with PDI antibody (Abcam, ab2792, Waltham, MA, USA) diluted 1:100 in 200 µL PBS-T for 10 min. Beads were again washed with PBS-T and incubated with 400 μL cell culture media for 1 h. Following a final wash with PBS-T, samples were eluted with 40 μL of 1× LDS (Thermo Fisher Scientific, Waltham, MA, USA). After heating samples at 70 °C for 10 min, equal volumes of samples were loaded into a 4–12% Bis-Tris gel (Thermo Fisher Scientific, Waltham, MA, USA) and run using 1× MOPS buffer (Thermo Fisher Scientific, Waltham, MA, USA). An iBlot2 (Thermo Fisher Scientific, Waltham, MA, USA) was used to transfer proteins to a 0.2 µm PVDF membrane (Thermo Fisher Scientific, Waltham, MA, USA) using Program P0. Blots were blocked at room temperature for 1 h using Rockland blocking buffer (VWR, Radnor, PA, USA). Primary PDI antibody (Abcam, ab2792, Waltham, MA, USA) diluted 1:500 was applied overnight at 4 °C and secondary goat anti-mouse IR680 antibody (LICOR, Lincoln, NE, USA) diluted 1:4000 was applied for 1 h at room temperature. Blots were scanned using an Odyssey scanner (LICOR, Lincoln, NE, USA). Data are presented as arbitrary density units (ADU) relative to control. 

### 4.7. MANF Homogeneous Time-Resolved Fluorescence (HTRF) Assay

The amount of MANF present in cell culture media samples was quantified using a MANF HTRF assay (Cisbio, Bedford, MA, USA) as per the manufacturer’s instructions and in accordance with previously published data [36,73]. Briefly, media samples diluted 1:2 in dilution buffer were incubated with anti-Human MANF-d2 antibody and anti-Human-Eu^3+^ cryptate antibody for 24 h. Fluorescence was measured at 665 nm and 620 nm using a BioTek Synergy H1 plate reader (Biotek/Agilent, Santa Clara, CA, USA) and sample readouts were compared to a MANF standard curve. Data are presented as MANF in ng/mL relative to control.

### 4.8. Esterase Assay

Extracellular esterase presence was measured as previously described [34,73,78]. Immediately before hyperthermia treatments began cells underwent a full media exchange into esterase assay media (150 mM NaCl, 5 mM KCl, 1 mM MgCl_2_, 20 mM HEPES, 1 mM CaCl_2_, and 1.9 g/L glucose). An interaction between esterases and a fluorescein di-(1-methylcyclopropanecarboxymethyl ether) produces fluorescence that was quantified by a BioTek Synergy H1 plate reader (excitation 465 nm/emission 528 nm) (Biotek/Agilent, Snata Clara, CA, USA). An equal volume of cell culture media and esterase substrate (100 µM in esterase assay media at pH 5) was transferred to a black-walled, clear-bottomed plate (PerkinElmer, Waltham, MA, USA) and fluorescence was measured after 1 h. Data are presented as raw fluorescent units (RFU) relative to control.

### 4.9. siRNA Transfection

SH-SY5Y cells (2.5 × 10^4^ cells/well, 96-well plate) stably expressing GLuc-ASARTDL or GLuc-Untagged were reverse transfected with 10 nM siRNA using Lipofectamine RNAiMAX (Thermo Fisher Scientific, Waltham, MA, USA). A full media exchange was performed 48 h after transfection and after a further 24 h incubation the cells were placed in hyperthermic or normothermic conditions for 24 h. The siRNAs used were from the Thermo Fisher Scientific Silencer Select Library (Waltham, MA, USA): KDELR1 (assay s548), KDELR2 (assay s21689), KDELR3 (assay s21690), and control (Cat. #4390843). This was performed in accordance with a previous publication [36]. 

### 4.10. Lentiviral Transduction

Lentiviral vectors expressing Myc- and FLAG- tagged KDEL receptors 1, 2, and 3 were previously described [33]. Viruses were titered using the Lenti-X p24 Rapid Titer Kit (Takara, Kusatsu, Japan). SH-SY5Y was transduced in 96-well plates at multiplicity of infection (MOI) of 2, incubated for 48 h, and then exposed to hyperthermic conditions as described above. This was performed in accordance with a previously published experimental timeline [36]. 

### 4.11. Real-Time qPCR

RNA was isolated from cells using a NucleoSpin RNA Plus kit (Macherey-Nagel, Allentown, PA, USA) according to the manufacturer’s protocol. Using iScript (Bio-Rad, Hercules, CA, USA) 500 ng of RNA was transcribed into cDNA in a 20 µL reaction. The cDNA was diluted 1:40 with DNase-free water. 5 µL of cDNA was assayed in duplicate in a 20 µL reaction composed of TaqMan Universal PCR Master Mix (Thermo Fisher Scientific, Waltham, MA, USA), 450 nM primers, and 100 nM probe. Real-time qPCR was performed using a C1000 Thermal Cycler CFX96 Real-Time System (Bio-Rad, Hercules, CA, USA) with a pre-incubation (50 °C for 10 s followed by photobleach, repeat 20×, 95 °C for 5 min) and amplification with 50 repeats (94 °C for 20 s, 60 °C for 1 min). All C_q_ values were normalized to the C_q_ for glyceraldehyde 3-phosphate dehydrogenase **(***GAPDH*). Two other reference genes, ubiquitin-conjugating enzyme 2i (*Ube2i*) and RNA polymerase II (*PRNAII*), were evaluated for use, but did not remain constant over treatment paradigms. Results are presented as the 2^-ddCq^ value ± upper and lower limits (limits calculated based on the standard deviation of delta Cq values). Data are presented as relative mRNA expression (arbitrary units) relative to control. The primer and probe sequences used were the following: human *ASNS*, ggattggctgccttttatcagg (forward), ggcttctttcagctgcttcaac (reverse), tggactccagcttggttgctgcc (FAM-labeled probe); human *BiP*, gttgtggccactaatggagatac (forward), ggagtttctgcacagctctattg (reverse), acgctggtcaaagtcttctccaccca (FAM-labeled probe); human *ERdj4*, gccatgaagtaccaccctg (forward), ccactagtaaaagcactgtgtc (reverse), ctgcaatctctctgaattttgcttcagc (FAM-labeled probe); human *PRNAII*, gcaccacgtccaatgacattg (forward), ggagccatcaaaggagatgac (reverse), acggcttcaatgcccagcaccg (HEX-labeled probe); human *Ube2i*, gtgtgcctgtccatcttagag (forward), gctgggtcttggatatttggttc (reverse), caaggactggaggccagccatcac (HEX-labeled probe); human KDELR1, tgctccttcaccacggtctg (forward), ggtgaagtcatgattgaccaggaacg (reverse), agttcctggtcgttcccacagccattctg (FAM-labeled probe); human *KDELR2*, ctatgccacagtgtacctgatc (forward), agagaaatcgtgattaactaaaaatgag (reverse), ctacgatggaaatcatgataccttccgag (FAM-labeled probe); human *KDELR3*, gaggtccaagtgctgcaagg (forward), cactgtaacataggcacagaggag (reverse), caccaggtacctggacctgttcaccaa (FAM-labeled probe) (Integrated DNA Technologies); human *GAPDH*, catcatccctgcctctactg (forward), cttgccacagccttggagc (reverse), ccagtgagcttcccgttca (HEX-labeled probe). The procedures as well as the primer and probe sequences used here are in accordance with previously published methods [36,73]. 

### 4.12. Quantification and Statistical Analysis

Results were analyzed using GraphPad Prism 9 (San Diego, CA, USA) or SAS/STAT 14 (Cary, NC, USA). The specific tests are described in the figure legends. Multiple wells from multi-welled plates were used in varied positions within the plates and results were reproduced in ≥2 independent experiments. Cell culture plates were randomly chosen for hyperthermia versus normal temperature exposure, but the investigator was not blinded to the treatments. Data was analyzed with Prism 9 using two-tailed Student’s t-test or two-way ANOVA with multiple comparisons tests (Dunnett’s, Sidak’s, and Tukey’s). Data was analyzed with SAS/STAT 14 using three-way ANOVA with slice decomposition (slice option in SAS’s GLM procedure). Data are presented as mean ± standard error of the mean (SEM) with error bars representing SEM unless otherwise stated. For mRNA expression data, results are presented as 2^-ddCq^ with the error bars showing the upper and lower limits calculated using the standard deviation (SD) for delta C_q_ values. Statistical tests of qPCR data were performed using delta C_q_. The sample size and significance for each figure are shown in the corresponding figure legend. 

## 5. Conclusions

Hyperthermia is a trigger of ER-resident protein secretion (ER exodosis), and both caffeine and MDMA further increase secretion. ER-resident proteins are necessary for intrinsic cellular processes, and the secretion of such proteins can negatively impact cellular functioning. Hyperthermia also increases UPR target gene expression, with MDMA causing further increases in *BiP* mRNA levels. Despite being IRE1α/XBP1 regulated genes, KDELR2 and KDELR3 were not increased in hyperthermia, but KDEL receptor knockdown led to increases in ER exodosis. Stabilizing ER calcium or increasing KDEL receptor expression can attenuate ER exodosis, indicating two possible therapeutic avenues for treating disruption of ER proteostasis caused by hyperthermia alone or in combination with MDMA and caffeine.

## Data Availability

Data are available within the article or Supplementary Materials or are available on request from the authors.

## Supplementary Materials

The following supporting information can be downloaded at: https://www.mdpi.com/article/10.3390/ijms23041974/s1.
