# Supplementary material for: Caffeine and MDMA (Ecstasy) Exacerbate ER Stress Triggered by Hyperthermia
Source: Int J Mol Sci. 2022 Feb 10;23(4):1974. doi: 10.3390/ijms23041974 (PMC8880705; doi:10.3390/ijms23041974)
Supplement: Supplementary file 1 [file ijms-23-01974-s001.zip › ijms-1577627-supplementary.pdf]

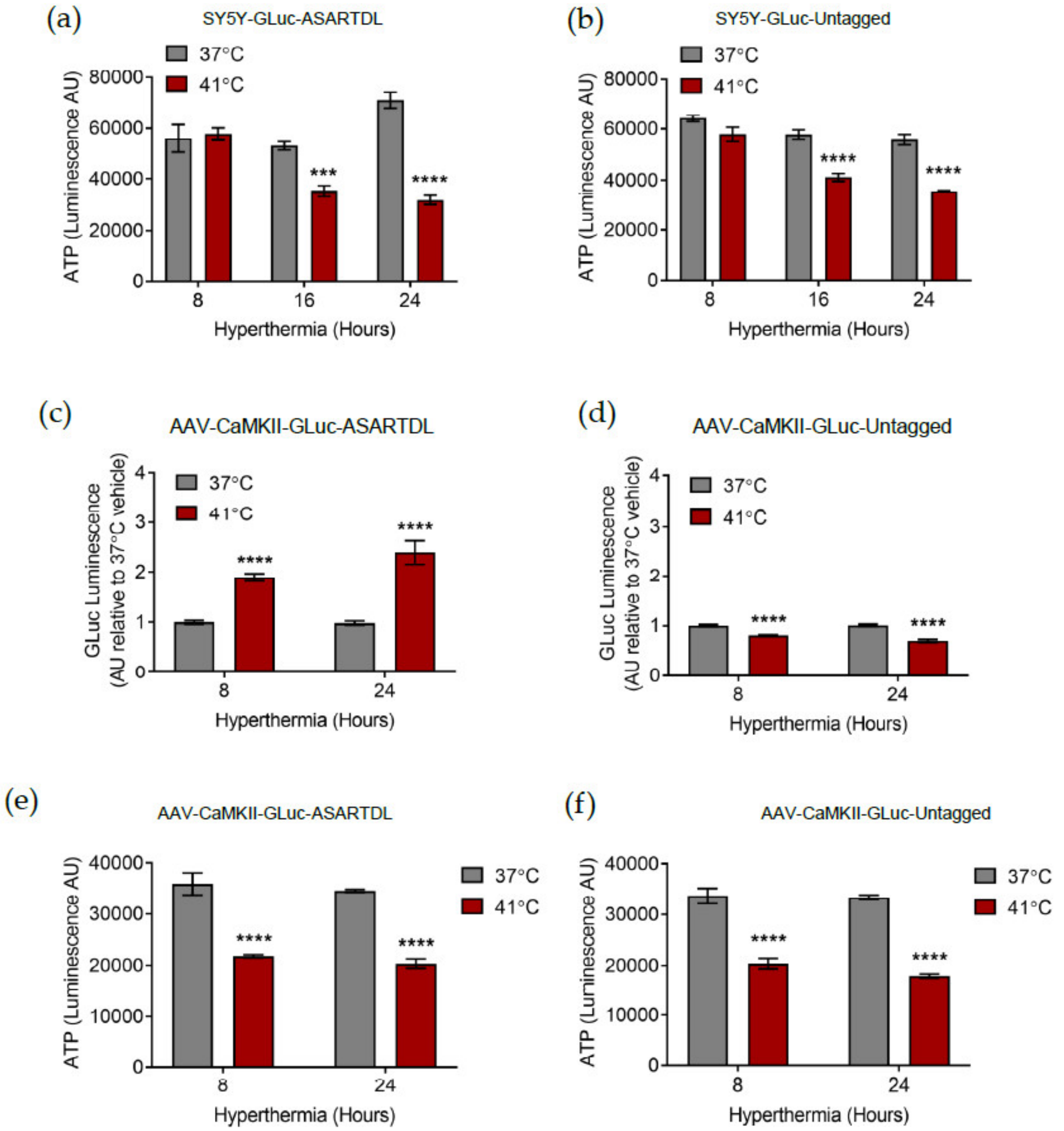


**Figure S1.** Hyperthermia increases GLuc-ASARTDL secretion and decreases cell metabolic activity. (**a-b**) ATP assay (arbitrary units: AU) of SH-SY5Y cells stably expressing (**a**) GLuc-ASARTDL or (**b**) GLuc-Untagged after an 8 h, 16 h, or 24 h incubation at 37°C or 41°C (mean ± SEM, n=6, two-way ANOVA with Sidak's multiple comparisons, ***p<0.001 and ****p<0.0001 37°C vs. 41°C). (**c-d**) GLuc (arbitrary units: AU) in the media from PCNs transduced with (**c**) GLuc-ASARTDL or (**d**) GLuc-Untagged after an 8 h or 24 h incubation at 37°C or 41°C (mean ± SEM, n=27, two-way ANOVA with Sidak's multiple comparisons, ****p<0.0001 37°C vs. 41°C). (**e-f**) ATP assay (arbitrary units: AU) of PCNs transduced with (**e**) GLuc-ASARTDL or (**f**) GLuc-Untagged after an 8 h or 24 h incubation at 37°C or 41°C (mean ± SEM, n=6, two-way ANOVA with Sidak's multiple comparisons, ****p<0.0001 37°C vs. 41°C).

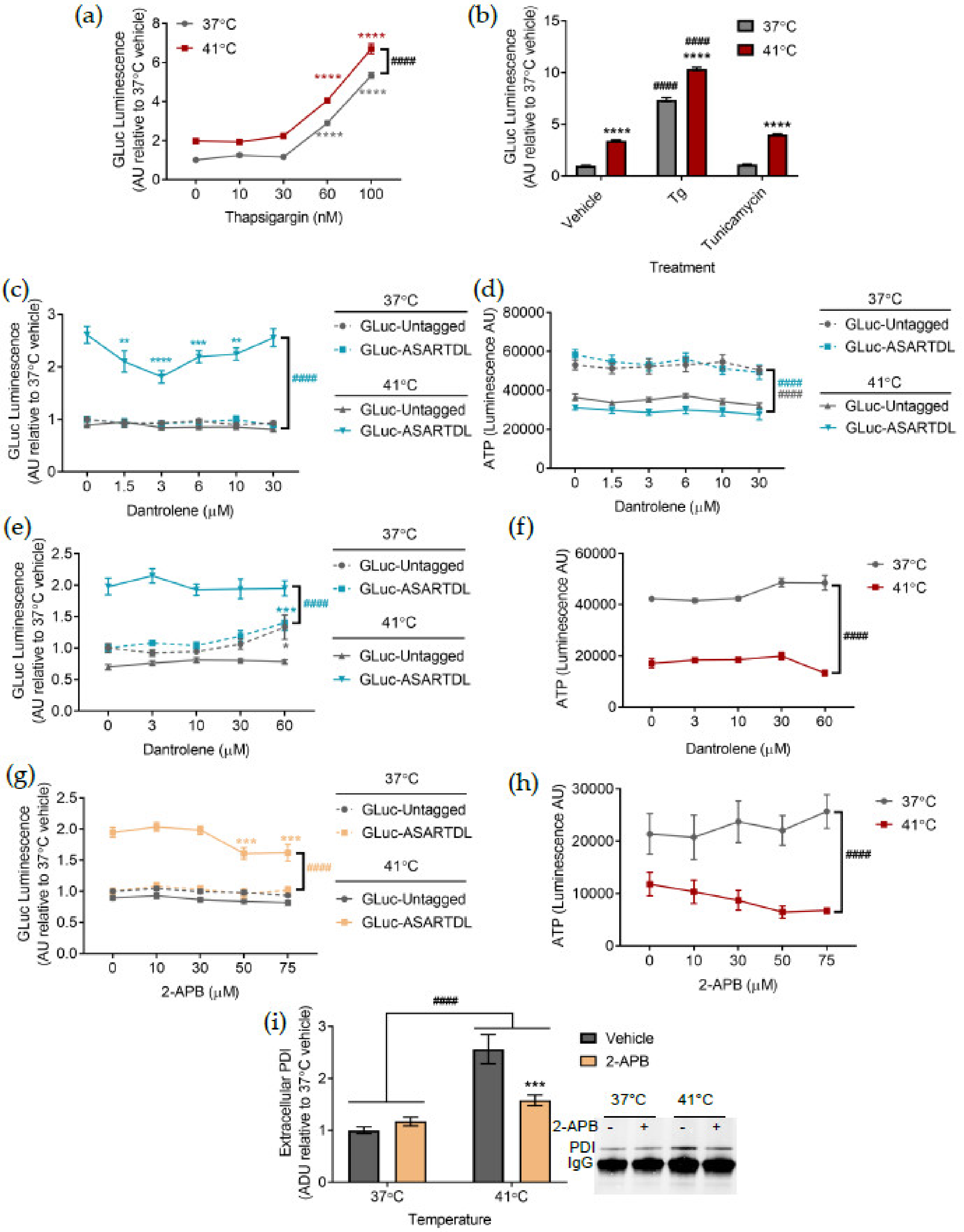


**Figure S2.** Modulation of ER calcium affects hyperthermia-induced ER exodosis. (**a**) GLuc (arbitrary units: AU) in the media from SH-SY5Y cells stably expressing GLuc-ASARTDL following treatment with vehicle or thapsigargin and a 24 h incubation at 37°C or 41°C (mean ± SEM, n=16, two-way ANOVA with Dunnett's multiple comparisons,

####p<0.0001 37°C vs. 41°C, ****p<0.0001 vehicle vs. thapsigargin). (**b**) GLuc (arbitrary units: AU) in the media from SH-SY5Y cells stably expressing GLuc-ASARTDL following treatment with vehicle, 200 nM thapsigargin, or 3 µg/mL tunicamycin treatment and a 24 h incubation at 37°C or 41°C (mean ± SEM, n=24, two-way ANOVA with Tukey's multiple comparisons, ****p<0.0001 37°C vs. 41°C, ####p<0.0001 vehicle vs. drug). (**c**) GLuc (arbitrary units: AU) in the media from SH-SY5Y cells stably expressing either GLuc-ASARTDL or GLuc-Untagged after a 16 h pre-treatment with dantrolene followed by a 24 h incubation at 37°C or 41°C (mean ± SEM, n≥9, two-way ANOVA with Dunnett's multiple comparisons, ####p<0.0001 37°C vs. 41°C, **p<0.01, ***p<0.001, and ****p<0.0001 vehicle vs. dantrolene) (**d**) ATP assay (arbitrary units: AU) of SH-SY5Y cells stably expressing GLuc-ASARTDL or GLuc-Untagged after a 16 h pre-treatment with dantrolene followed by a 24 h incubation at 37°C or 41°C (mean ± SEM, n=9, two-way ANOVA with Dunnett's multiple comparisons, ####p<0.0001 37°C vs. 41°C). (**e**) GLuc (arbitrary units: AU) in the media from PCNs transduced with either GLuc-ASARTDL or GLuc-Untagged after a 30 min pre-treatment with dantrolene followed by a 24 h incubation at 37°C or 41°C (mean ± SEM, n=6, two-way ANOVA with Dunnett's multiple comparisons, ####p<0.0001 37°C vs. 41°C, ***p<0.001 vehicle vs. dantrolene). (**f**) ATP assay (arbitrary units: AU) of PCNs transduced with GLuc-ASARTDL after a 30 min pre-treatment with dantrolene followed by a 24 h incubation at 37°C or 41°C (mean ± SEM, n=9, two-way ANOVA with Dunnett's multiple comparisons, ####p<0.0001 37°C vs. 41°C). (**g**) GLuc (arbitrary units: AU) in the media from PCNs transduced with either GLuc-ASARTDL or GLuc-Untagged after a 30 min pre-treatment with 2-APB followed by a 24 h incubation at 37°C or 41°C (mean ± SEM, n=18, two-way ANOVA with Dunnett's multiple comparisons, ####p<0.0001 37°C vs. 41°C, ***p<0.001 vehicle vs. 2-APB). (**h**) ATP assay (arbitrary units: AU) of PCNs transduced with GLuc-ASARTDL after a 30 min pre-treatment with 2-APB followed by a 24 h incubation at 37°C or 41°C (mean ± SEM, n=9, two-way ANOVA with Dunnett's multiple comparisons, ####p<0.0001 37°C vs. 41°C). (**i**) Fold change in arbitrary density units (ADU) of immunoprecipitated PDI (representative blot shown) in media from PCNs pre-treated with vehicle or 50 µM 2-APB for 30 min then incubated for 24 h at 37°C or 41°C (mean ± SEM, n=6, two-way ANOVA with Sidak's multiple comparisons, ####p<0.0001 37°C vs. 41°C, ***p<0.001 vehicle vs. 2-APB).

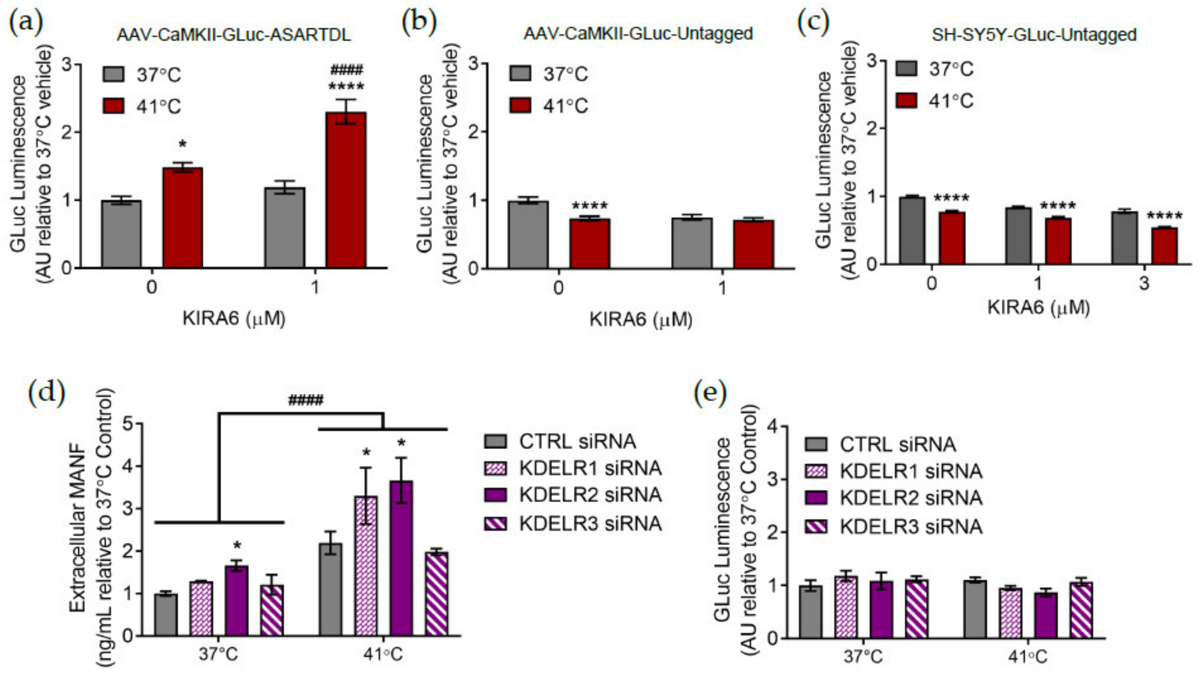


**Figure S3.** Hyperthermia associated changes related to the UPR and KDEL receptors. (**a-b**) GLuc (arbitrary units: AU) in the media from PCNs transduced with (**a**) GLuc-ASARTDL or (**b**) GLuc-Untagged after a 1 h pre-treatment with vehicle or 1 µM KIRA6 followed by a 24 h incubation at 37°C or 41°C (mean ± SEM, n=15, two-way ANOVA with Tukey's multiple comparisons, *p<0.05 and ****p<0.0001 37°C versus 41°C, ####p<0.0001 vehicle vs. KIRA6). (**c**)

GLuc (arbitrary units: AU) in the media from SH-SY5Y cells stably expressing GLuc-Untagged after a 1 h pre-treatment with vehicle or KIRA6 (1 µM or 3 µM) followed by a 24 h incubation at 37°C or 41°C (mean ± SEM, n=48, two-way ANOVA with Tukey's multiple comparisons, ****$p<0.0001$ 37°C vs. 41°C). (**d**) MANF in media following transfection of SH-SY5Y with 10 nM KDEL receptor siRNA and a 24 h incubation at 37°C or 41°C (mean ± SEM, n=6, two-way ANOVA with Dunnett's multiple comparisons, $^{\#\#\#\#}p<0.0001$ 37°C vs. 41°C, *$p<0.05$ and **$p<0.01$ control vs. KDELR siRNA). (**e**) GLuc (arbitrary units: AU) in media following transfection of SH-SY5Y cells stably expressing GLuc-Untagged with 10 nM KDEL receptor siRNA and a 24 h incubation at 37°C or 41°C (mean ± SEM, n=12, two-way ANOVA with Dunnett's multiple comparisons).

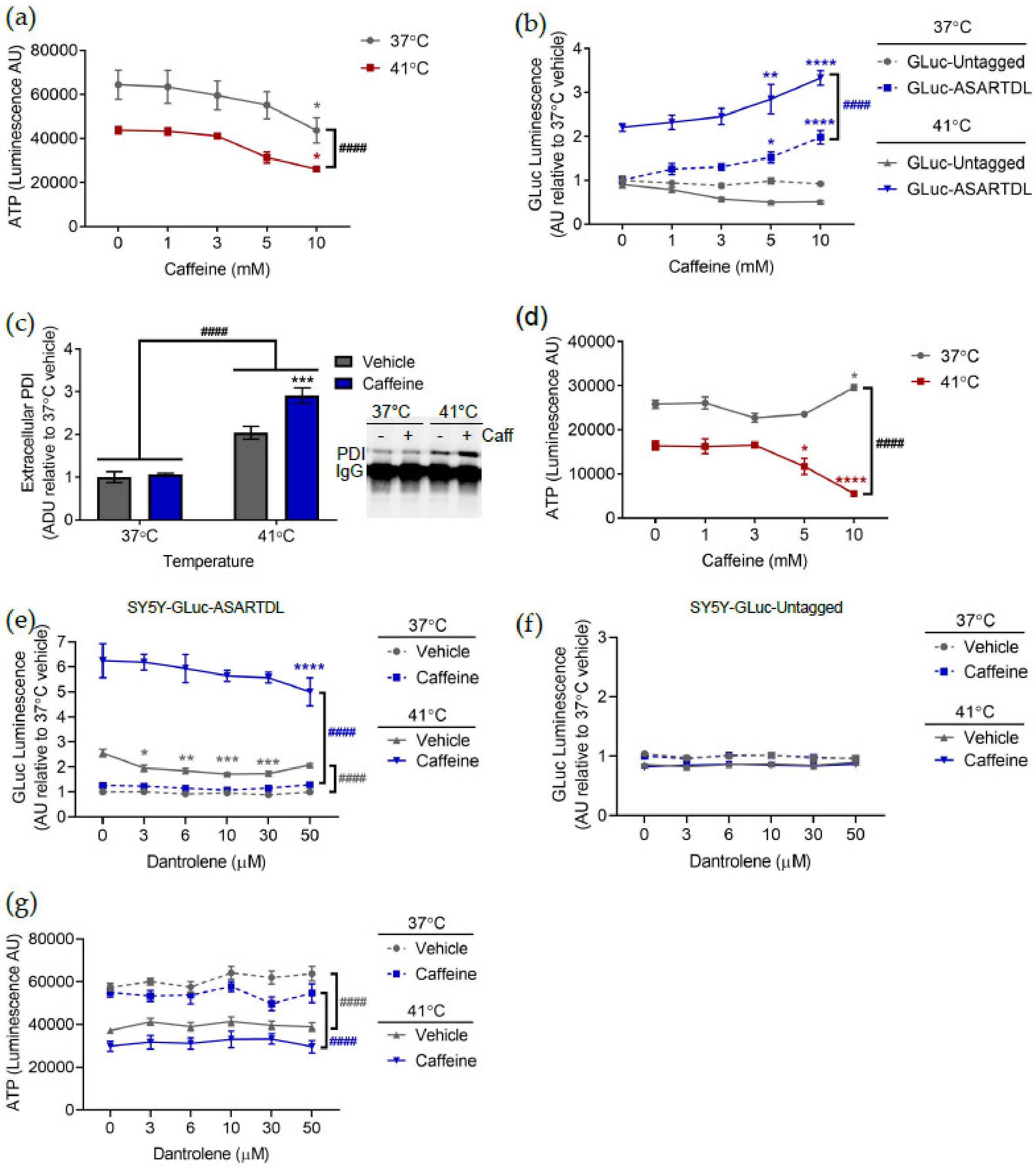

**Figure S4**. Caffeine associated changes in hyperthermia-induced ER. (**a**) ATP assay (arbitrary units: AU) of SH-SY5Y cells stably expressing GLuc-ASARTDL after treatment with vehicle or caffeine and a 24 h incubation at 37°C or 41°C (mean ± SEM, n=6, two-way ANOVA with Dunnett's multiple comparisons, $^{\#\#\#\#}p<0.0001$ 37°C versus 41°C, $^{*}p<0.05$ vehicle vs. caffeine). (**b**) GLuc (arbitrary units: AU) in the media from PCNs transduced with GLuc-ASARTDL or GLuc-Untagged after treatment with vehicle or caffeine and a 24 h incubation at 37°C or 41°C (mean ± SEM, n=12, two-way ANOVA with Dunnett's multiple comparisons, $^{\#\#\#\#}p<0.0001$ 37°C vs. 41°C, $^{*}p<0.05$, $^{**}p<0.01$, $^{****}p<0.0001$ vehicle vs. caffeine). (**c**) Fold change in arbitrary density units (ADU) of immunoprecipitated PDI (representative blot shown) in media from PCNs treated with vehicle or 5 mM caffeine and incubated for 24 h at 37°C or 41°C (mean ± SEM, n=6, two-way ANOVA with Sidak's multiple comparisons, $^{\#\#\#\#}p<0.0001$ 37°C vs. 41°C, $^{***}p<0.001$ vehicle vs. caffeine). (**d**) ATP assay (arbitrary units: AU) of PCNs transduced with GLuc-ASARTDL after treatment with vehicle or caffeine and a 24 h incubation at 37°C or 41°C (mean ± SEM, n=6, two-way ANOVA with Dunnett's multiple comparisons, $^{\#\#\#\#}p<0.0001$ 37°C vs. 41°C, $^{*}p<0.05$ and $^{****}p<0.0001$ vehicle vs. caffeine). (**e**) GLuc (arbitrary units: AU) in the media from SH-SY5Y cells stably expressing GLuc-ASARTDL after a 16 h pre-treatment with dantrolene followed by treatment with 5 mM caffeine and a 24 h incubation at 37°C or 41°C (mean ± SEM, n=16, two-way ANOVA with Dunnett's multiple comparisons, $^{\#\#\#\#}p<0.0001$ 37°C vs. 41°C, $^{*}p<0.05$, $^{**}p<0.01$, $^{***}p<0.001$, and $^{****}p<0.0001$ vehicle vs. dantrolene). (**f**) GLuc (arbitrary units: AU) in the media from SH-SY5Y stably expressing GLuc-Untagged after a 16 h pre-treatment with dantrolene followed by treatment with 5mM caffeine and a 24 h incubation at 37°C or 41°C (mean ± SEM, n=16, two-way ANOVA with Dunnett's multiple comparisons). (**g**) ATP assay (arbitrary units: AU) of SH-SY5Y cells stably expressing GLuc-ASARTDL after a 16 h pre-treatment with dantrolene followed by treatment with 5 mM caffeine and a 24 h incubation at 37°C or 41°C (mean ± SEM, n=16, two-way ANOVA with Dunnett's multiple comparisons, $^{\#\#\#\#}p<0.0001$ 37°C vs. 41°C).

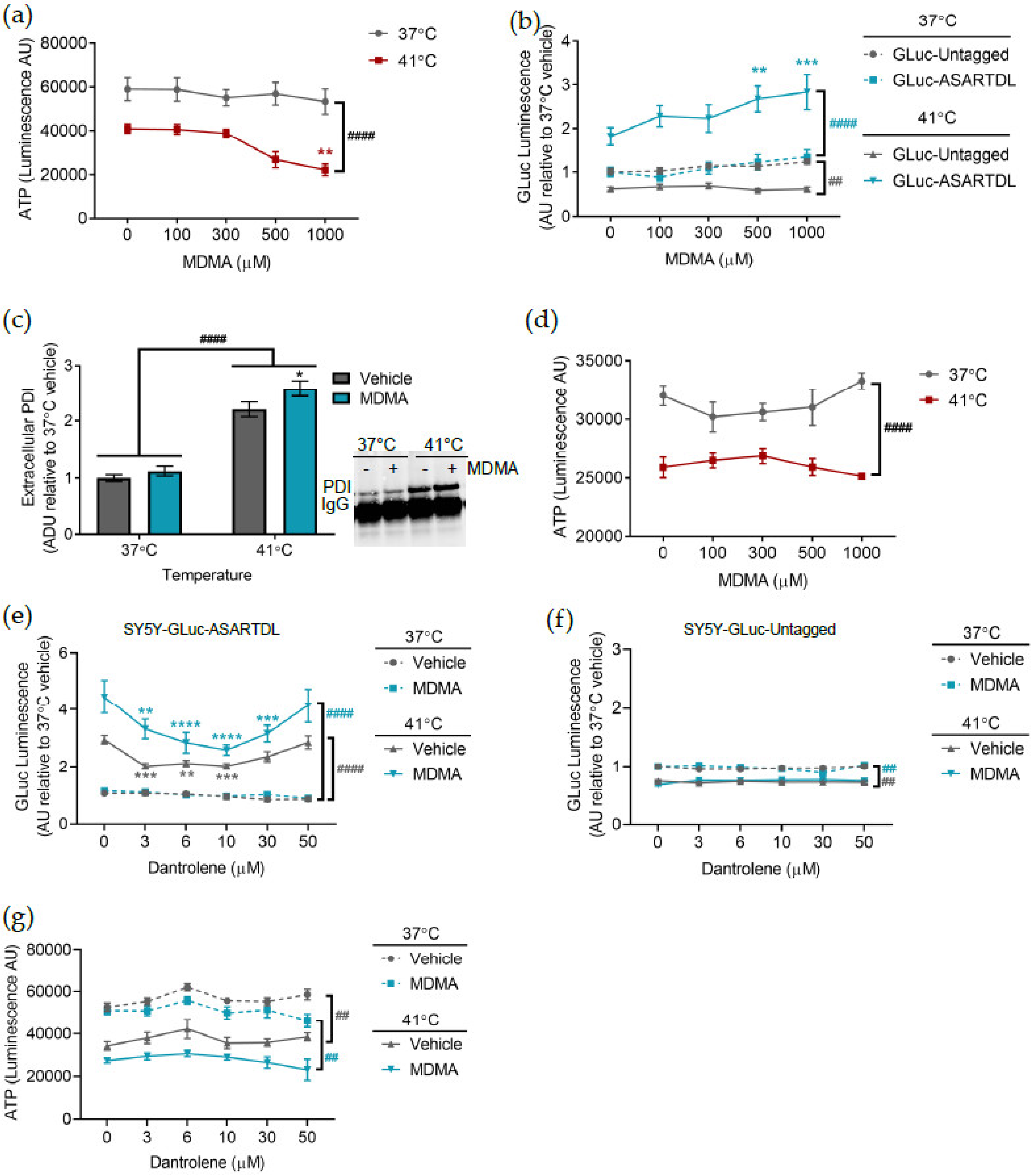


**Figure S5.** MDMA associated changes in hyperthermia-induced ER exodosis. (**a**) ATP assay (arbitrary units: AU) of SH-SY5Y cells stably expressing GLuc-ASARTDL after treatment with vehicle or MDMA and a 24 h incubation at 37°C or 41°C (mean ± SEM, n=6, two-way ANOVA with Dunnett's multiple comparisons, ####p<0.0001 37°C versus 41°C, **p<0.01 vehicle vs. MDMA). (**b**) GLuc (arbitrary units: AU) in the media from PCNs transduced with GLuc-ASARTDL or GLuc-Untagged after treatment with vehicle or MDMA and a 24 h incubation at 37°C or 41°C (mean ± SEM, n=12, two-way ANOVA with Dunnett's multiple comparisons, ##p<0.01 and ####p<0.0001 37°C vs. 41°C, **p<0.01 and ***p<0.001 vehicle vs. MDMA). (**c**) Fold change in arbitrary density units (ADU) of immunoprecipitated PDI (representative blot shown) in media from PCNs treated with vehicle or 500 μM MDMA and incubated for 24 h at 37°C or 41°C (mean ± SEM, n=6, two-way ANOVA with Sidak's multiple comparisons, ####p<0.0001 37°C vs. 41°C,

*p<0.05 vehicle vs. MDMA). (**d**) ATP assay (arbitrary units: AU) of PCNs transduced with GLuc-ASARTDL after treatment with vehicle or MDMA and a 24 h incubation at 37°C or 41°C (mean ± SEM, n=6, two-way ANOVA with Dunnett's multiple comparisons, ####p<0.0001 37°C vs. 41°C). (**e**) GLuc (arbitrary units: AU) in the media from SH-SY5Y cells stably expressing GLuc-ASARTDL after a 16 h pre-treatment with dantrolene followed by treatment with 1 mM MDMA and a 24 h incubation at 37°C or 41°C (mean ± SEM, n=16, two-way ANOVA with Dunnett's multiple comparisons, ####p<0.0001 37°C vs. 41°C, **p<0.01, ***p<0.001, ****p<0.0001 vehicle vs. dantrolene). (**f**) GLuc (arbitrary units: AU) in the media from SH-SY5Y stably expressing GLuc-Untagged after a 16 h pre-treatment with dantrolene followed by treatment with 1 mM MDMA and a 24 h incubation at 37°C or 41°C (mean ± SEM, n=16, two-way ANOVA with Dunnett's multiple comparisons, ##p<0.01 37°C vs. 41°C). (**g**) ATP assay (arbitrary units: AU) of SH-SY5Y cells stably expressing GLuc-ASARTDL after a 16 h pre-treatment with dantrolene followed by treatment with 1 mM MDMA and a 24 h incubation at 37°C or 41°C (mean ± SEM, n=16, two-way ANOVA with Dunnett's multiple comparisons, ##p<0.01 37°C vs. 41°C).

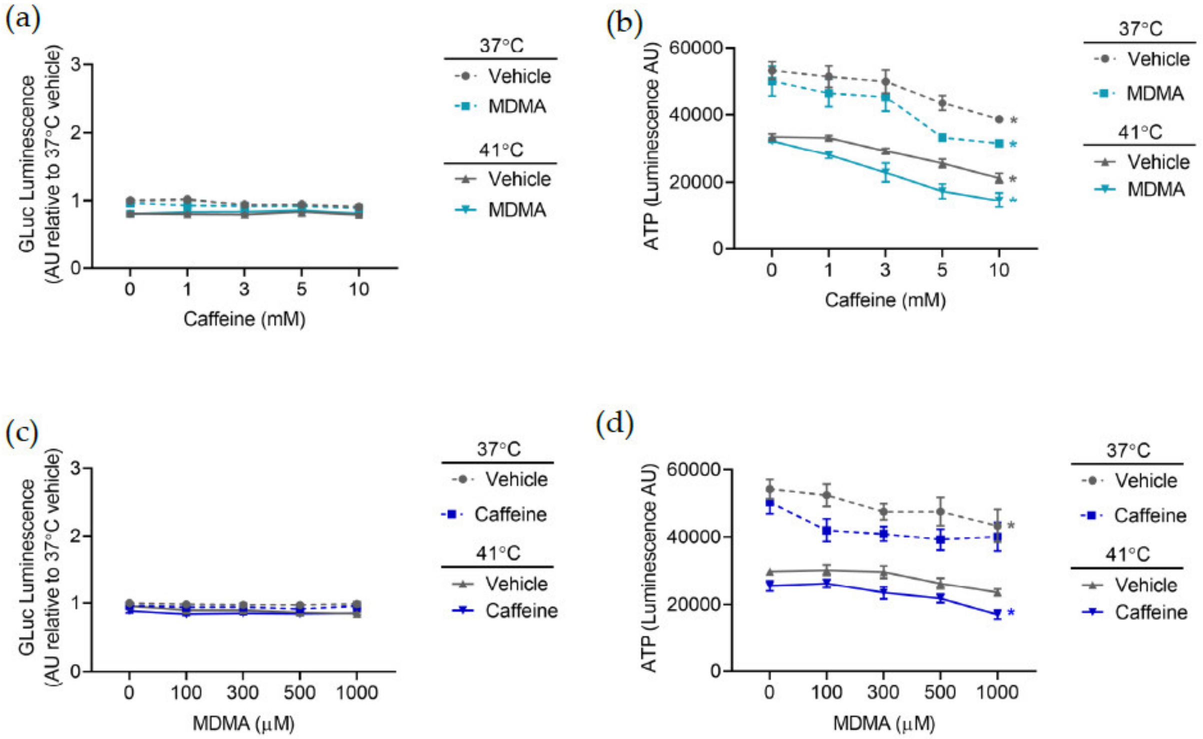


**Figure S6**. Caffeine and MDMA in combination affect cellular ATP, but not GLuc-Untagged secretion in hyperthermic conditions. (**a**) GLuc (arbitrary units: AU) in the media from SH-SY5Y cells stably expressing GLuc-Untagged after treatment with vehicle or 500 μM MDMA in combination with a dose response of caffeine and a 24 h incubation at 37°C or 41°C (mean ± SEM, n=9, three-way ANOVA with Slice decomposition). (**b**) ATP assay (arbitrary units: AU) of SH-SY5Y-GLuc-ASARTDL cells treated with vehicle or 500 μM MDMA in combination with a dose response of caffeine and a 24 h incubation at 37°C or 41°C (mean ± SEM, n=9, three-way ANOVA with Slice decomposition, p<0.001 37°C vs. 41°C, *p<0.05 vehicle vs. caffeine). (**c**) GLuc (arbitrary units: AU) in the media from SH-SY5Y cells stably expressing GLuc-Untagged after treatment with vehicle or 1 mM caffeine in combination with a dose response of MDMA and a 24 h incubation at 37°C or 41°C (mean ± SEM, n=9, three-way ANOVA with Slice decomposition). (**d**) ATP assay (arbitrary units: AU) of SH-SY5Y-GLuc-ASARTDL cells treated with vehicle or 1 mM caffeine in combination with a dose response of MDMA and a 24 h incubation at 37°C or 41°C (mean ± SEM, n=9, three-way ANOVA with Slice decomposition, p<0.001 37°C vs. 41°C, *p<0.05 vehicle vs. MDMA).
